# Prostate tumor-induced angiogenesis is blocked by exosomes derived from menstrual stem cells through the inhibition of reactive oxygen species

**DOI:** 10.18632/oncotarget.9852

**Published:** 2016-06-06

**Authors:** Francisca Alcayaga-Miranda, Paz L. González, Alejandra Lopez-Verrilli, Manuel Varas-Godoy, Carolina Aguila-Díaz, Luis Contreras, Maroun Khoury

**Affiliations:** ^1^ Laboratory of Nano-Regenerative Medicine, Faculty of Medicine, Universidad de los Andes, Santiago, Chile; ^2^ Cells for Cells, Santiago, Chile; ^3^ Laboratory of Reproductive Biology, Faculty of Medicine, Universidad de los Andes, Santiago, Chile; ^4^ Servicio de Anatomía Patológica, Clínica Universidad de los Andes, Santiago, Chile; ^5^ Consorcio Regenero, Chilean Consortium for Regenerative Medicine, Santiago, Chile

**Keywords:** mesenchymal stem cells, exosomes, tumor angiogenesis, ROS pathway, prostate cancer

## Abstract

Mesenchymal stem cells (MSCs) secrete exosomes that are capable of modifying the tumor environment through different mechanisms including changes in the cancer-cell secretome. This activity depends on their cargo content that is largely defined by their cellular origin. Endometrial cells are fine regulators of the angiogenic process during the menstrual cycle that includes an angiostatic condition that is associated with the end of the cycle. Hence, we studied the angiogenic activity of menstrual stem cells (MenSCs)-secreted exosomes on prostate PC3 tumor cells. Our results showed that exosomes induce a reduction in VEGF secretion and NF-κB activity. Lower reactive oxygen species (ROS) production in exosomes-treated cells was detected by the DCF method, suggesting that the inhibition of the intracellular ROS impacts both NF-κB and VEGF pathways. We confirmed using tubule formation and plug transplantation assays that MenSCs-exosomes suppress the secretion of pro-angiogenic factors by the PC3 cells in a ROS-dependent manner. The inhibition of the tumor angiogenesis and, consequently, the tumor growth was also confirmed using a xenograft mouse model. Additionally, the anti-tumoral effect was associated with a reduction of tumor hemoglobin content, vascular density and inhibition of VEGF and HIF-1α expression. Importantly, we demonstrate that the exosomes anti-angiogenic effect is specific to the menstrual cell source, as bone marrow MSCs-derived exosomes showed an opposite effect on the *VEGF* and *bFGF* expression in tumor cells. Altogether, our results indicate that MenSCs-derived exosomes acts as blockers of the tumor-induced angiogenesis and therefore could be suitable for anti-cancer therapies.

## INTRODUCTION

Vascular endothelial growth factor (VEGF) is the major pro-angiogenic factor in physiological and pathological conditions, which is often regulated by hypoxia-inducible factor (HIF-1α), matrix metalloproteases (MMPs), and a wide range of other transcription factors and metabolic regulators, such as the E26 transformation-specific (ETS) family of transcription factors and reactive oxygen species (ROS) [1]. ROS are essentially required to induce physiological angiogenesis, but uncontrolled, continuous ROS production promotes pathological angiogenesis operating mainly on the VEGF signaling pathway [2]. Several studies have demonstrated that exogenous ROS stimulate the induction of VEGF expression in various cell types, such as endothelial cells, smooth muscle cells and macrophages, whereas VEGF induces endothelial cell migration and proliferation through an increase of intracellular ROS [2, 3]. In the tumor mass, tumor and stromal cells produce substantial amounts of ROS [4] and the endogenous ROS production by the tumor cells regulates angiogenesis and tumor development through HIF-1α and VEGF [5, 6].

Mesenchymal stem cells (MSCs) are multipotent cells that exhibit a marked tropism for tumors [7, 8] and display controversial angiogenic properties: while some reports showed that MSCs support tumor angiogenesis [9–11], other studies observed a range of anti-angiogenic properties [12–14]. This can be explained by many reasons, such as the complexity of the different MSCs origins and their specific properties on different cancer cell types [15–17], as a consequence of the specific crosstalk between tumor cells, tumor microenvironment and MSCs, and by the MSCs-derived factors produced within the tumor [18]. Some of the mechanisms involved in the inhibition of tumor angiogenesis by MSCs include the production of MSCs-derived ROS that induce cytotoxicity in endothelial cells [13], and the down-regulation of different angiogenic signaling pathways, including PDGF/PDGFR [14] and VE-Cadherin/β-catenin axis [19].

Exosomes are membrane vesicles of endocytic origin ranging in size from 30-150 nanometers (nm) [20, 21]. They are emerging as key mediators in intercellular communications through horizontal transfer of information via their molecular cargo, which includes proteins, DNAs, messenger RNAs and micro(mi)RNAs, that could trigger specific intracellular cascades that affect the gene expression of the recipient cells [21–23]. In the context of tumor angiogenesis, exosomes derived from different MSCs origins also have shown opposite effects. It has been reported that MSCs-secreted exosomes exert both pro- and anti-angiogenic effects mediating the up- and down-regulation of *VEGF* expression in cancer cells, respectively [24, 25]. Although it is not completely understood, these opposing results could be explained by the fact that exosomes derived from different sources of MSCs bear the specific molecular signature of their cells of origin, and hence, enclose different molecules which deliver different information into their microenvironments [15, 26].

Based on the knowledge that physiological angiogenesis occurs mainly during the female reproductive cycle [27], we believe that resident stem cells are fine regulators of the angiogenic process. In fact, endometrial stromal cells exhibit remarkable changes in their angiogenic status throughout the menstrual cycle, from high angiogenic activity associated with rapid endometrial expansion at the beginning of the cycle, followed by an angiostatic condition that is associated with the end of the cycle [28]. Therefore, we focused our study on menstrual stem cells (MenSCs), isolated from menstrual blood. In this context, although MenSCs have been previously reported as multipotent cells with a potent angiogenic effect [29, 30], the angiogenic response of MenSCs or its paracrine signals, specifically through exosomes, in a tumor context remains unknown.

Here, we demonstrate for the first time that the uptake of MenSCs-derived exosomes by tumor cells results in a reduction of ROS production, which serves as a signal to modulate VEGF expression in cancer cells, and consequently inhibit neovascularization and tumor development. We further demonstrate the specificity of this response, as in contrast to MenSCs, BMSCs-derived exosomes failed to induce a similar anti-angiogenic effect.

## RESULTS

### Characterization of MenSCs-derived exosomes

Consistently with previous reports [29, 31, 32, 37], MenSCs express CD105, CD44, CD73, CD90 and HLA-ABC, but showed negative expression for CD45, CD34, CD14 and HLA-DR (Figure S1 A). Also, mesodermal lineage induction showed positive specific staining for fat, bone and cartilage differentiation (Figure S1 B).

MenSCs-derived exosomes were successfully purified from the MenSCs-CM by serial centrifugation as was previously described [34]. Electron microscopy (EM) analysis of the exosomes revealed a typical round-shaped appearance and size of ~94 ± 2 nm (Figure S2 A). The size as measured by nanoparticle tracking analysis (NTA) was ~134.1 ± 6.2 nm (Figure S2 B). In accordance with previous reports [26, 38], immunoblotting showed positive expression of HSP90, HSP70 and CD63, which were enriched in comparison with the cell lysate, while the mitochondrial markers cytochrome C was absent in the purified exosome fraction (Figure S2 C).

### MenSCs-derived exosomes inhibit angiogenic factors in prostate cancer cells

**Figure 1:**
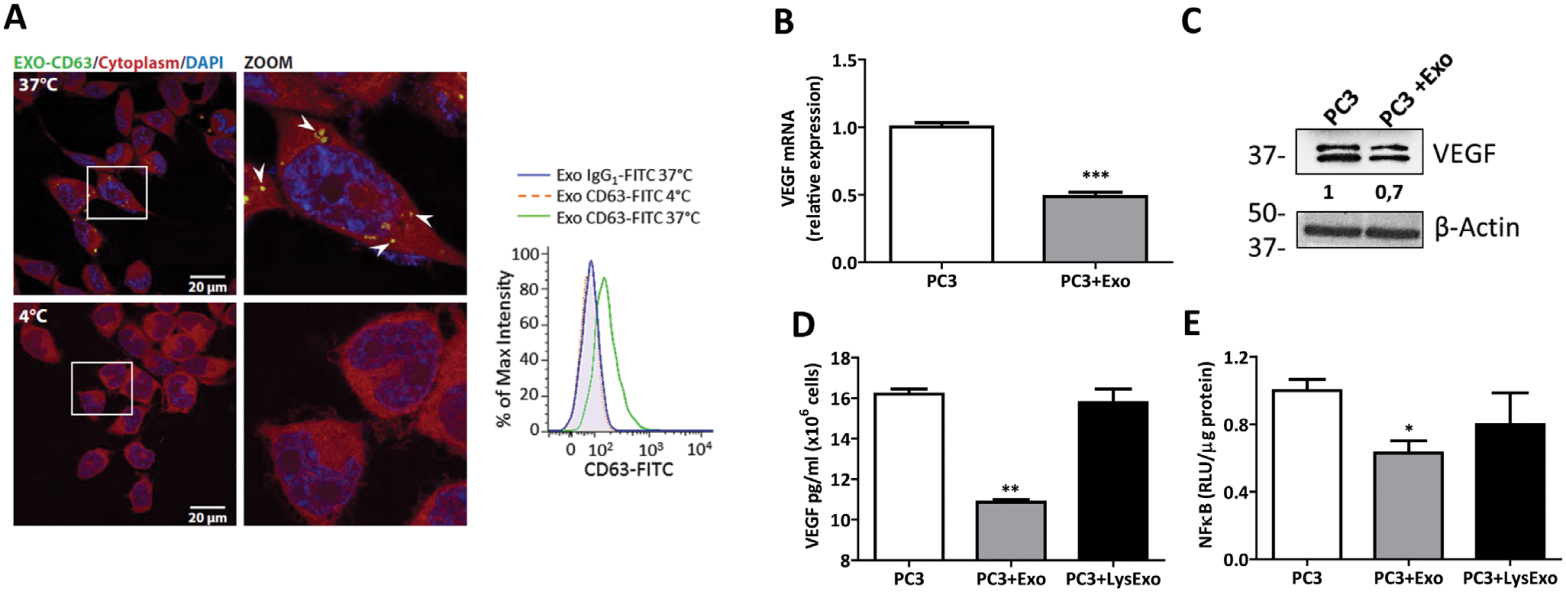
MenSCs-derived exosomes down-regulate *VEGF* and *FGF* expression and NF-κB activity **A.** PC3 cells were incubated with immuno-labeled MenSCs-derived exosomes (20 μg) for 3 hours at either 37°C or 4°C and uptake of exosomes by PC3 cells was assessed. Exosomes internalization (white arrows) was visualized with confocal microscopy (left panel) and flow cytometry (right panel). **B-E.** PC3 cells were incubated in the absence or presence of MenSCs-derived exosomes or lysed exosomes for 36 hours and their effects on *VEGF* and NF-κB were determined. Relative expression level of *VEGF* were assessed by qRT-PCR (B); Protein level of VEGF was determined by western-blot (C); VEGF secretion was measured by ELISA (D); NF-κB activity was assessed by luciferase reporter assay (E). Data are presented as mean values ± SE. Western blotting results were evaluated by densitometry, corrected with respect to β-actin expression, and expressed relative to the value obtained with the corresponding control (arbitrarily set as 1). Equal protein loading was assessed by anti–β-actin immunoblotting. Abbreviations: MenSCs, menstrual derived mesenchymal stem cells; Exo, exosomes; LysExo, lysed exosomes; VEGF, vascular endothelial growth factor; FGF, fibroblast growth factor; NF-κB, nuclear factor- kappa B; SE, standard error.

To assess the putative interactions between MenSCs-derived exosomes and human prostate adenocarcinoma PC3 cells, the uptake of exosomes by PC3 cells was studied using FACS and confocal microscopy. As shown in Figure 1A (left panel), anti-CD63-FITC labeled exosomes were localized in the cytoplasm of PC3 cells revealing the internalization of the exosomes. Consistently with other reports [39, 40], no green fluorescence signal was detected after incubation at 4°C, indicating that exosomes internalization by PC3 cells was mediated by an energy-dependent process. The quantification of these data showed that PC3 cells contain 28.25 ± 2.85% of green fluorescent exosomes based on the percentage of max intensity of the population peaks after 3 hours of incubation; meanwhile a decrease in temperature to 4°C induced a reduction of 98.6 ± 0.005% in the uptake of exosomes by PC3 cells (Figure 1A, right panel).

MSCs-derived exosomes have been reported to both promote [25] and abrogate [24] tumor angiogenesis. To delineate the tumor angiogenic effects of MenSCs-secreted exosomes, mRNA levels of *VEGF-A* (hereafter referred as VEGF) and *bFGF* were determined in PC3 cells. As shown in Figure 1B and S3, MenSCs-derived exosomes down-regulated the mRNA levels of *VEGF* and *bFGF* in PC3 cells (p < 0.001). This was further confirmed by the reduction of the protein level of VEGF in comparison with untreated cells (Figure 1C). To confirm the anti-angiogenic effect of exosomes, secreted levels of VEGF were determined in the PC3-CM. The lysed exosomes condition was used as control to confirm the exosomes-specific effect. Consistently with the downregulation of the *VEGF* mRNA levels, a ~1.5 fold (p < 0.01) decrease in VEGF secretion was detected in the PC3-CM treated with MenSCs-derived exosomes. In contrast, the lysed exosomes condition had no effect on the secretion of VEGF, corroborating an exosomes-specific response (Figure 1D).

Since the NF-κB transcription factor is involved in the regulation of *VEGF* and is constitutively activated in human prostate cancer cells [41], we investigated the effect of the MenSCs-derived exosomes on NF-κBactivity. As shown in Figure 1E, exosomes treatment decreased the NF-κBactivity compared with untreated PC3 cells (p < 0.05), while the addition of lysed exosomes had no effect in NF-κBactivity. These results indicate that the observed inhibition of NF-κBactivity was mediated by exosomes.

### MenSCs-derived exosomes decrease angiogenesis through inhibition of the ROS pathway

**Figure 2:**
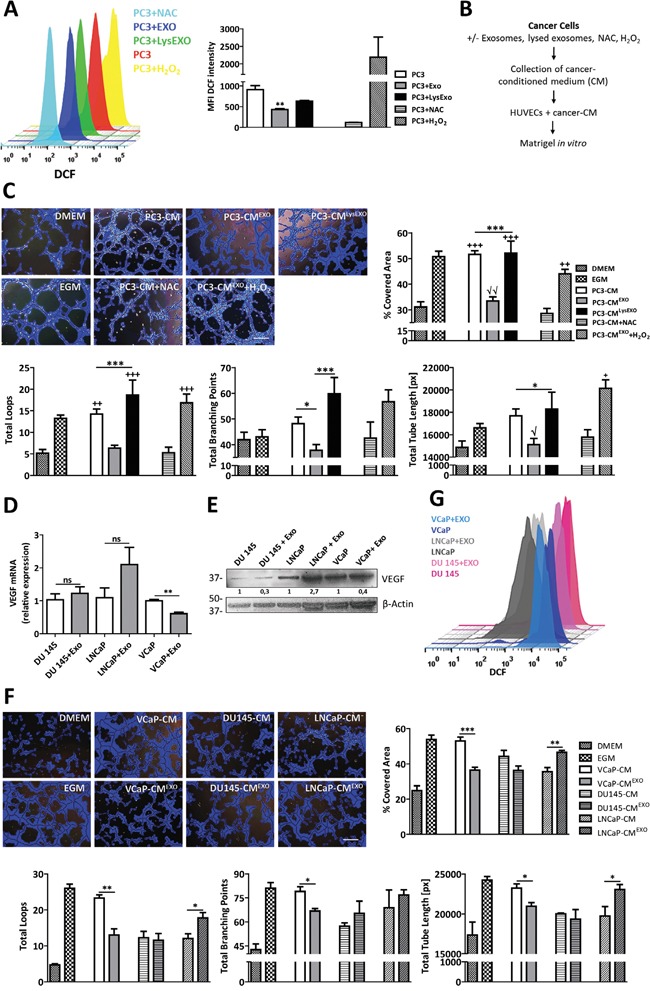
Inhibition of ROS by MenSCs-derived exosomes reduce angiogenesis *in vitro* **A.** PC3 cells were treated with or without exosomes or lysed exosomes and assayed for ROS production by FACS using the fluorescent dye DCFDA. NAC and H_2_O_2_ were used as negative and positive control, respectively. Representative histograms show ROS generation in each experimental condition. Graph represents the mean values ± SE of the MFI of DCF. *, respect to PC3. **B-C.** HUVEC were resuspended in the CM derived from PC3 cells treated with exosomes, exosomes plus H_2_O_2_, lysed exosomes, NAC, or untreated, and seeded in matrigel (B); Representative images show tube formation after 5 hours. Graphs represent the quantitative analysis of the angiogenic potential (C). **D-G.** A panel of prostate cancer cell lines were incubated in the absence or presence of MenSCs-derived exosomes for 36 hours and their effect on *VEGF*, tube-like structures formation and ROS was determined. Relative expression levels of *VEGF* was assessed by qRT-PCR (D); Protein level of VEGF was determined by western-blot (E); Representative images show tube formation after 5 hours. Graphs represent the quantitative analysis of the angiogenic potential (F); ROS generation by FACS using the fluorescent dye DCFDA (G). Data are presented as mean values ± SE. Western blotting results were evaluated by densitometry, corrected with respect to β-actin expression, and expressed relative to the value obtained with the corresponding control (arbitrarily set as 1). Equal protein loading was assessed by anti–β-actin immunoblotting. Scale bar: 200 μm. *, respect to PC3-CM^EXO^; √, respect to PC3-CM^EXO^+H_2_O_2_; +, respect to PC3-CM+NAC. Abbreviations: MenSCs, menstrual derived mesenchymal stem cells; EXO, exosomes; CM, conditioned medium; LysEXO, lysed exosomes; DMEM, Dulbecco's modified Eagle's medium; EGM, endothelial growth medium; NAC, N-acetylcysteine; ROS, reactive oxygen species; DCFDA, 2′, 7′–dichlorofluorescin diacetate; DCF, 2′, 7′–dichlorofluorescin; FACS, fluorescence-activated cell sorting; MFI, mean fluorescent intensity; SE, standard error.

PC3 cells spontaneously produce ROS which are involved in the malignant phenotype of prostate cancer cells [42]. In a report by Xia et al [5], ROS were shown to regulate angiogenesis and tumor growth through VEGF. In addition, Otsu et al [13] described that MSCs-derived ROS cause capillary degeneration and subsequently inhibition of tumor angiogenesis. Hence, we investigated the possibility that ROS signaling might be involved in exosomes-regulated angiogenesis through the modulation of VEGF in PC3 cells. For this, endogenous ROS production in prostate cancer cells was determined by FACS using the cell-permeable fluorescent probe DCFDA. As shown in Figure 2A, internalization of MenSCs-derived exosomes resulted in decreased ROS production in comparison with the basal expression levels in untreated PC3 cells (p < 0.01). These reduced levels were comparable to the ROS-inhibitor (NAC) condition. The lysed exosomes showed a reduced effect on ROS expression, suggesting that the ROS modulation was in part mediated by exosomes.

To confirm that the reduction of endogenous ROS in PC3 cells is involved in the inhibition of prostate tumor angiogenesis, the formation of HUVECs into tube-like structures on Matrigel was evaluated in the presence of conditioned medium from PC3 cells previously pre-treated under 3 different conditions including exosomes treatment (PC3-CM^EXO^), lysed exosomes treatment (PC3-CM^LysEXO^) or untreated (PC3-CM) (Figure 2B). As expected, in an endothelial growth medium (EGM-2) used as a positive control, HUVECs formed an extensive network of tube-like structures. The tube forming ability was lost when HUVECs were cultured in non-endothelial growth medium (DMEM). As shown in Figure 2C, image analysis of the tube formation revealed that PC3-CM or PC3-CM^LysEXO^ prompted a more extensive network of tube-like structures in comparison with the PC3-CM^EXO^ or NAC alone. Induction of an oxidative stress through the addition of H_2_O_2_ treatment to PC3-CM^EXO^ suppressed the anti-angiogenic effect of MenSCs-derived exosomes, suggesting that the effect was ROS-dependent. Quantitative analysis of Matrigel assessment was performed by determining percentage of covered area, total loops, total branching points and total tube length in the tube-like structures. As expected, a reduction in all the measured parameters was observed in the PC3-CM^EXO^ group with respect to the PC3-CM group. This effect was reverted in the presence of PC3-CM^LysEXO^ or in the addition of H_2_O_2_. Interestingly, quantitative analysis revealed that PC3-CM^EXO^ decreased these angiogenic parameters in almost similar levels as the antioxidant NAC.

To enquire whether the observed anti-angiogenic effect extends to other prostate cancer cells, MenSCs-exosomes were tested on VCaP, DU145 and LNCaP cell lines. While the exosomes achieved the downregulation of VEGF in VCaP cells at both mRNA and protein levels (Figure 2D and 2E), no effect was detected in DU145 and LNCaP cells. Consequently, DU145-CM previously incubated with MenSCs-derived exosomes (DU145-CM^EXO^) had no effect in the formation of tube-like structures in the matrigel *in vitro* assay (Figure 2F), while VCaP-CM^EXO^ showed a strong reduction in the tube-like structures developed in comparison with untreated cells (VCaP-CM). Image analysis in VCaP condition showed a lower percentage of covered area (p < 0.001), number of loops and branching points (p < 0.01; p < 0.05), and smaller tube length (p < 0.05) after treatment with exosomes. In contrast, LNCaP-CM^EXO^ stimulated an extensive network of tube-like structures in comparison with LNCaP-CM. To determine whether these different observations were mediated by ROS, endogenous ROS levels were determined in the cell lines. The exosomes treatment resulted in a reduction of the ROS level in VCaP cells, while an increase of ROS were determined in LNCaP cells. Unexpectedly, the treatment with MenSCs-exosomes in DU145 cells decreased the endogenous ROS level (Figure 2G). Taken together, these data suggest that MenSCs-derived exosomes impact the prostate cancer cells angiogenic effect in a cell line specific manner.

### Reduction of tumor angiogenesis by exosomes is dependent on both MSCs origins and tumor types

**Figure 3:**
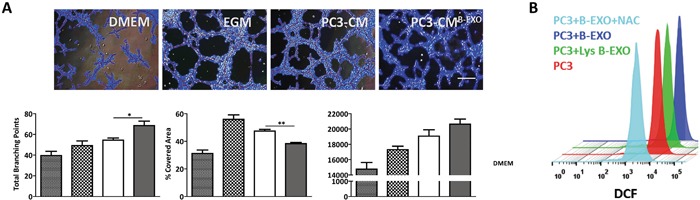
BMSCs-derived exosomes increase tumor angiogenesis *in vitro* **A.** HUVECs were resuspended in CM from PC3 cells treated with BMSC-derived exosomes or untreated, and seeded in matrigel. Representative images show tube formation after 5 hours. Graphs represent quantitative analysis of the angiogenic potential. Data are presented as mean values ± SE. **B.** PC3 cells were treated with or without BMSC-derived exosomes or lysed exosomes, and assayed for ROS production by FACS using the fluorescent dye DCFDA. Representative histograms show endogenous ROS production in each experimental condition. Data are presented as mean values ± SE. Scale bar: 200 μm. Abbreviations: BMSCs, bone marrow derived mesenchymal stem cells; EXO, exosomes; CM, conditioned medium; DMEM, Dulbecco's modified Eagle's medium; EGM, endothelial growth medium; ROS, reactive oxygen species; NAC, N-acetylcysteine; DCFDA, 2′, 7′–dichlorofluorescein diacetate; DCF, 2′, 7′–dichlorofluorescein; FACS, fluorescence-activated cell sorting; SE, standard error.

To determine whether the observed effects of exosomes on tumor angiogenesis were specific to MenSCs or transversal to exosomes derived from other sources of MSCs, BMSCs-derived exosomes were isolated and their angiogenic effects on cancer cells assessed. In contrast to MenSC-derived exosomes, the addition of PC3-CM previously incubated with BMSCs-derived exosomes (PC3-CM^B-EXO^) stimulated the formation of tube-like structures in comparison with the untreated PC3-CM. A higher number of branching points (p < 0.05), longer tube length (p < 0.05) and smaller % of covered area (p < 0.01) were also developed in following treatment with PC3-CM^B-EXO^ with respect to PC3-CM, suggesting that the angiogenic effect of exosomes is dependent of the source of MSCs they are derived from (Figure 3A). To confirm the role of ROS in BMSCs-exosomes effect, we determined the endogenous ROS levels in PC3 cells. As shown in Figure 3B, while BMSCs-exosomes increase the ROS levels in PC3 cells, the lysed exosomes had no effect on ROS production, indicating that ROS modulation was mediated by exosomes. The addition of NAC to the BMSCs-derived exosomes completely inhibited the fluorescent signal, revealing the specificity of the ROS staining.

**Figure 4:**
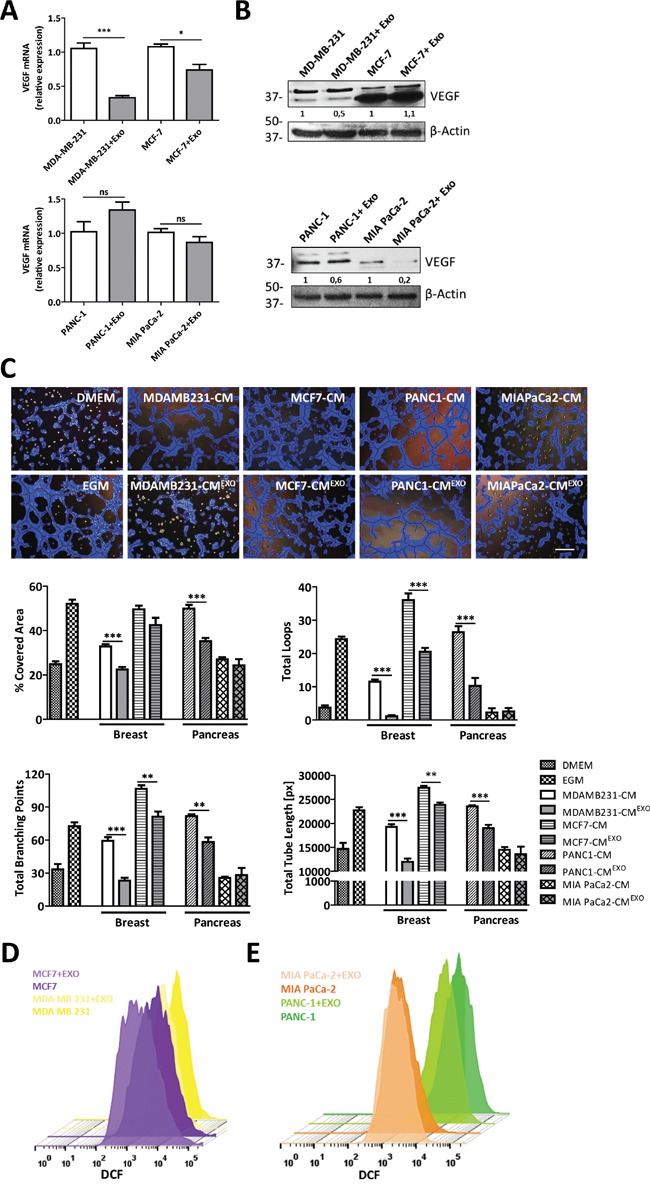
MenSCs-derived exosomes inhibition of tumor angiogenesis is cancer cell-type dependent **A-B.** Breast (MDA-MB-231 and MCF-7) and pancreatic (MIA PaCa-2 and PANC-1) cancer cell lines were incubated in the absence or presence of MenSCs-derived exosomes for 36 hours and their effects on *VEGF* were determined. Relative expression levels of *VEGF* was assessed by qRT-PCR (A) and western-blot (B). **C.** HUVECs were resuspended in CM from tumor cells treated with MenSCs-derived exosomes or untreated, and seeded in matrigel. Representative images show tube formation after 5 hours. Graphs represent quantitative analysis of the angiogenic potential. Data are presented as mean values ± SE. Western blot results were evaluated by densitometry, corrected with respect to β-actin expression, and expressed relative to the value obtained with the corresponding control (arbitrarily set as 1). Equal protein loading was assessed by anti–β-actin immunoblotting. Scale bar: 200 μm. **D-E.** Cancer cells were treated with or without MenSCs-derived exosomes and assayed for ROS production by FACS using the fluorescent dye DCFDA. Representative histograms show endogenous ROS production in each experimental condition. Abbreviations: EXO, exosomes; CM, conditioned medium; DMEM, Dulbecco's modified Eagle's medium; EGM, endothelial growth medium; ROS, reactive oxygen species; DCFDA, 2′, 7′–dichlorofluorescein diacetate; DCF, 2′, 7′–dichlorofluorescein; FACS, fluorescence-activated cell sorting; ns, non-significant; SE, standard error.

To define whether the anti-angiogenic effect of MenSCs-exosomes is cancer type-dependent, mRNA levels of *VEGF* and *bFGF* were also determined in two breast (MDA-MB-231 and MCF-7) and pancreatic (PANC-1 and MiaPaCa-2) cancer cell lines following culture with the exosomes. The treatment of breast and pancreatic cancer cells with MenSCs-derived exosomes showed inconsistent effects on the expression of angiogenic proteins. As shown in Figure 4A, although MenSCs-exosomes down-regulated the *VEGF* mRNA levels in breast cancer cell lines (p < 0.001 MDA-MB-231 and p < 0.05 MCF-7), no effect was observed in pancreatic cancer cells. Likewise, exosomes treatment induced an up-regulation of *bFGF* mRNA-level in MDA-MB-231 (p < 0.05) and PANC-1 (p < 0.05) cell lines, without any detectable effect on MCF-7 and MIA PaCa-2 cell lines (Figure S4). Furthermore, exosomes treatment decreased the VEGF protein level in MDA-MB-231 cells, whereas the VEGF level was unchanged in MCF-7 cells. As well, a reduction in VEGF was detected in both pancreatic cancer cell lines (Figure 4B). To test the functional effects of these expression changes in the panel of cancer cell lines, an *in vitro* angiogenesis assay was carried out (Figure 4C). Consistently with the reduction of *VEGF* in breast cancer cells, image analysis of tube formation revealed a reduction in the tube-like structures in MDA-MB-231-CM^EXO^ and MCF-7-CM^EXO^ in comparison with untreated cells. As expected, the quantitative analysis revealed that a reduced number of loops and branching points, coupled with lower covered area, and shorter tube length were developed after treatment with MenSCs-derived exosomes. In pancreatic cancer cells, both MIA PaCa-2-CM and MIA PaCa-2-CM^EXO^ showed no effect in the formation of tube-like structures, probably due to the low level of pro-angiogenic factors secreted by MIA PaCa-2 cells [43]. Unexpectedly, PANC-1-CM^EXO^ displayed a strong reduction in the formation of tube-like structures respect to PANC-1-CM, suggesting that exosomes may be modulating other angiogenic proteins, such as PDGF, angiopoietin 1 and angiopoietin 2, in PANC-1 cells. In contrast, the reduction of the breast cancer cells angiogenic profiles after treatment with exosomes was related to a reduction in their endogenous ROS levels (Figure 4D). In pancreatic cancer, ROS analysis revealed that in PANC-1 cells the treatment with exosomes resulted in a decreased ROS production in comparison with untreated cells. In MIA PaCa-2 cells, endogenous ROS levels were unchanged after exosomes treatment (Figure 4E).

### Reduction of tumor angiogenesis is specifically mediated by exosomes

**Figure 5:**
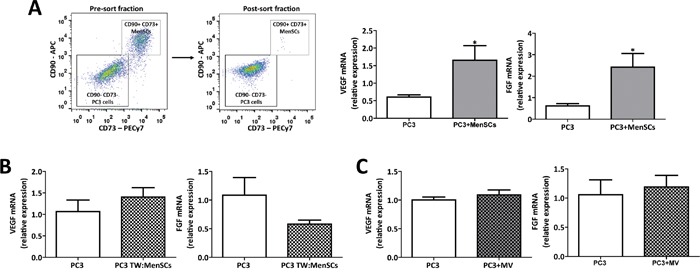
MenSCs-derived exosomes specifically decrease angiogenesis in prostate tumor cells **A.** PC3 cells were co-cultured with MenSCs for 36 hours and then separated by sorting. Relative expression levels of *VEGF* and *bFGF* in PC3 cells (CD73^−^/CD90^−^) were determined by qRT-PCR. **B.** PC3 cells were culture in a transwell system with MenSCs for 36 hours. Relative expression levels of *VEGF* and *bFGF* in PC3 cells were determined by qRT-PCR. **C.** PC3 cell were incubated with MenSCs-derived MVs for 36 hours. Relative expression levels of *VEGF* and *bFGF* were evaluated by qRT-PCR. Data are presented as mean values ± SE. Abbreviations: MenSCs, menstrual derived mesenchymal stem cells; TW, transwell; MV, microvesicles; SE, standard error.

We further aimed at understanding whether modulation of tumor angiogenesis is restricted to exosomes or whether it can also be mediated by MenSCs through their: (a) direct cell-to-cell contact (b) total secretome, or (c) microvesicular fraction. For this, mRNA levels of *VEGF* and *bFGF* were determined in PC3 cells cultured in the 3 different conditions including: direct contact with MenSCs and subsequently separated by cell sorting; separated by a transwell system; or treated with MenSCs-derived MVs. Conversely with the effects observed previously with the exosomes, MenSCs up-regulated the mRNA levels of *VEGF* and *bFGF* in PC3 cells (p < 0.05) (Figure 5A), while the total secretome and its microvesicular fraction showed no difference in mRNA level of *VEGF* and *bFGF* when compared to the control conditions (Figure 5B–5C), indicating that the observed modulation of tumor angiogenesis is an exosomes-specific effect.

### MenSCs-derived exosomes inhibit the tumoral angiogenic activity *in vivo*

**Figure 6:**
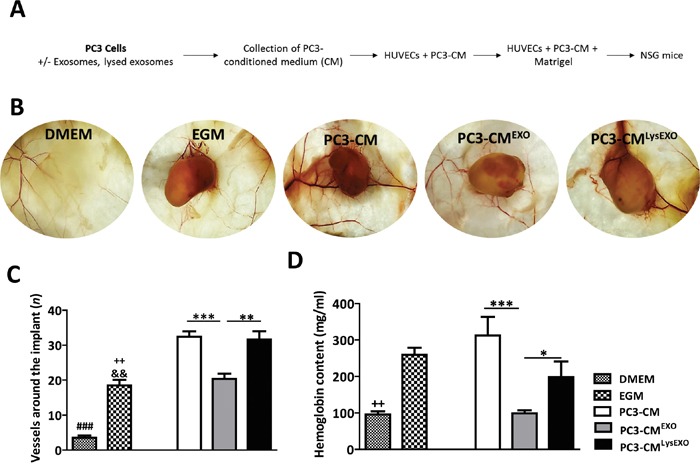
MenSCs-derived exosomes inhibit angiogenesis *in vivo* PC3 cells were incubated in absence or presence of exosomes or lysed exosomes for 36 hours and the CM was collected and mixed with HUVEC cells in matrigel to perform the angiogenesis assay *in vivo* (PC3-CM, *n*=8; PC3-CM^EXO^, *n*=8; PC3-CM^LysEXO^, *n*=6). The matrigel mixed with DMEM (*n*= 4) or EGM (*n*= 4) were used as negative and positive control, respectively. **A.** Diagram of the experimental design. **B.** Representative images of matrigel in NSG mice. **C.** Quantification of blood vessels around the matrigel implants. **D.** Hemoglobin content of the matrigel implants. Data represent mean ± SE. Scale bar: 200 μm. Abbreviations: MenSCs, menstrual derived mesenchymal stem cells; EXO, exosomes; CM, conditioned medium; LysEXO, lysed exosomes; DMEM, Dulbecco's modified Eagle's medium; EGM, endothelial growth medium; NSG, NOD scid gamma.

To confirm that MenSCs-derived exosomes inhibit the angiogenesis *in vivo*, Matrigels mixed with HUVECs previously resuspended in the PC3-CM, PC3-CM^EXO^ and PC3-CM^LysEXO^ were implanted into animals (Figure 6A). For controls, EGM-matrigel and DMEM-matrigel groups were prepared. As expected, profound inhibition of extra- and intra-plug angiogenesis were observed in the PC3-CM^EXO^ matrigel group in comparison with the PC3-CM matrigel group; in accordance with previous results, PC3-CM^LysEXO^ partially reverted the anti-angiogenic activity of the exosomes (Figure 6B). Image analysis reflected that PC3-CM^EXO^ decreased the number of vessels converging toward the implants with respect to PC3-CM (p < 0.001) and PC3-CM^LysEXO^ (p < 0.05) (Figure 6C). In addition, hemoglobin determination in matrigel plugs revealed a reduction in the hemoglobin levels contained in the PC3-CM^EXO^ matrigel group respect to PC3-CM (p < 0.001) and PC3-CM^LysEXO^ matrigel groups (p < 0.01) (Figure 6D).

**Figure 7:**
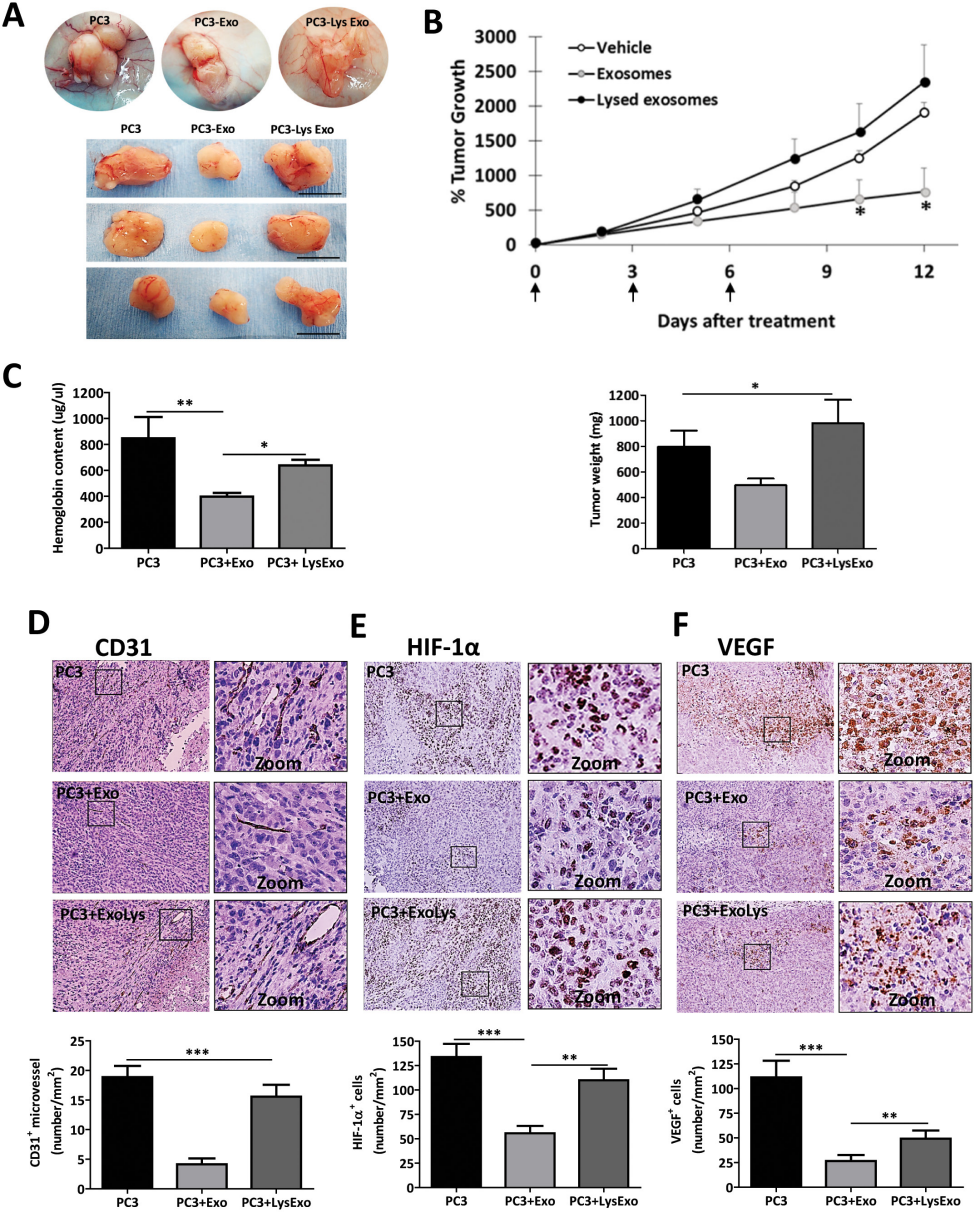
MenSCs-derived exosomes decrease angiogenesis and tumor growth *in vivo* PC3 cells (1.5×10^6^) were implanted subcutaneously in mice and when the tumor volume reached ~80 mm^3^, three injections of exosomes (*n*=12), lysed exosomes (*n*=8), and vehicle (*n*=14) were performed at defined time points (arrows in B). **A.** Representative images of tumors after treatments. Scale bar: 1 cm; **B.** Percentage of tumor growth and weight at day 12 after treatment; **C.** Hemoglobin content of the tumor tissue. **D-F.** CD31, HIF-1α and VEGF immunohistochemical staining and quantification. Images are shown at 40x. Data are presented as mean values ± SE. Abbreviations: MenSCs, menstrual derived mesenchymal stem cells; EXO, exosomes; LysEXO, lysed exosomes; VEGF, vascular endothelial growth factor; HIF-1α, hypoxia-inducible factor 1; SE, error standard.

To define the effects of MenSCs-derived exosomes on tumor angiogenesis and tumor growth, mice carrying PC3 tumors were injected thrice intratumorally with exosomes, lysed exosomes or vehicle. As expected, an inhibition of extra- and intra-tumor angiogenesis was observed following the exosomes treatment which induced decreased tumor growth with respect to vehicle and lysed exosomes injections (p < 0.05) (Figure 7A–7B). The quantification of the tissue hemoglobin content revealed a reduction in their level in the exosomes-inoculated group with respect to vehicle (p < 0.05) and lysed exosomes (p < 0.01) injections groups (Figure 7C). The analysis of CD31^+^ blood vessel in the tumor sections showed a lower vascular density in exosomes-inoculated group in comparison with vehicle (p < 0.001) and lysed exosomes (p < 0.001) injection groups (Figure 7D). HIF-1α, which is regulated at the protein level by oxygen dependent enzymes and, hence, allows for tissue hypoxia detection, showed nuclear positive staining in all the tumor tissues from the three groups but with a less prominent intensity in exosomes-inoculated tumors in comparison with vehicle (p < 0.001) and lysed exosomes-inoculated tumors (p < 0.01) (Figure 7E). This was coupled by a lower positive staining of VEGF positive staining observed in exosomes-inoculated tumors in comparison to the vehicle (p < 0.001) and lysed exosomes-treated (p < 0.01) tumor sections (Figure 7F).

## DISCUSSION

The initial event that we observed after MenSCs-derived exosomes internalization, process mediated by energy-dependent manner, was a change in the PC3 tumor cell-derived angiogenic-secretome, specifically a reduction in VEGF level. Since lysed exosomes had no effect on tumor-cell VEGF secretion, our results indicate that the effect of exosomes was mediated by molecules included in the exosomal cargo. Although it has been described that MSCs-secreted exosomes induce opposite effects on the angiogenic tumor secretome [24, 25], these paradoxical observations are probably related to the fact that MSCs-derived exosomes bear the specific molecular signatures of their cells of origin [26] and by the specific interaction between exosomes and recipient cells. Taking into consideration the over-expression of NF-κB that contributes to the up-regulation of *VEGF* mRNA expression resulting in the VEGF-induced angiogenesis through in many tumors (42, 43), including prostate cancer [41], we focused the study on looking at the effect of MenSCs-exosomes on NF-κB activity to elucidate the possible mechanism behind the modulation of the tumor cell secretome. Our results show that exosomes were involved in the reduction of NF-κB activity, an effect that was not observed after the incubation with lysed exosomes, indicating the specificity of the exosomal cargo effect. Although it has previously been reported that BMSCs-conditioned media, which contains a heterogeneous population of factors including exosomes, downregulates the mRNA levels of NF-κB in hepatoma and breast cancer cells [46], our observation represents the first report regarding the effect of MSCs-derived exosomes on NF-κB activity in tumor cells.

Several types of cancer cells are characterized by high levels of ROS production, which are involved in human carcinogenesis, tumor growth and angiogenesis [1, 2, 5, 6, 47]. Since ROS impact both NF-κB and VEGF signaling pathways in cancer cells [2, 5, 6, 13, 48, 49], we furthered the investigation towards evaluating the effect of exosomes on endogenous ROS production in tumor cells. Our findings showed that the exosomes induced a lower ROS production in PC3 cells, although the exact mechanism behind this effect remains unclear. However, the observation that lysed exosomes mitigates the exosomes effect point at a partial role of the exosomal cargo in ROS modulation. These results confirm previous findings where ROS was shown to be directly implicated in the regulation of the tumor angiogenesis [5, 13]. Using the CM from untreated or exosomes-treated PC3 cells, we evaluated the potential of HUVECs to form capillary-like structures in matrigel *in vitro*. Our results show that exosomes inhibit the angiogenic properties of the tumor cell-derived secretome, an effect that was not observed with lysed exosomes. Interestingly, the addition of H_2_O_2_ intended to abrogate the suppression of ROS production, blocked the anti-angiogenic property of exosomes in PC3 cancer cells, suggesting that exosomes modulate tumor angiogenesis in a ROS-dependent manner. Nonetheless, the variable exosomes effects observed on angiogenesis in a panel of prostate tumor cell lines suggest that their mechanisms of action is cell type dependent. Although more studies are necessary to identify the biological mechanism of this dichotomy, our observations point clearly at the role of MenSCs-exosomes in ROS signaling pathway.

The functional experiments in two different murine models demonstrate that MenSC-secreted exosomes also exert an important anti-angiogenic effect *in vivo*. In the angiogenesis plug assay, we confirmed that CM derived from exosomes-treated PC3 cells had reduced angiogenic properties, resulting in a lower number of vessels around the plugs and also, importantly, in the hemoglobin concentration inside the plugs. The anti-angiogenic efficacy via intratumoral administration was also evaluated in PC3-bearing mice. Treatment with three exosomes injections significantly reduced tumor angiogenesis and, consequently, tumor growth in comparison with the control and lysed exosomes groups. Our findings show that the anti-tumoral effect of exosomes was associated with a reduction in the tumor hemoglobin content, lower vascular density and loss of VEGF and HIF-1α tissue expression, which probably is a consequence of the lower ROS production induced by MenSC-derived exosomes. In fact, a previous report showed that endogenous ROS regulated the HIF-1α and *VEGF* expression in cancer cells, hence controlling the angiogenesis and tumor development *in vivo* [5]. In line with our observations, Lee and colleagues showed that in a breast tumor model murine BMSCs-secreted exosomes suppress angiogenesis by down-regulating *VEGF* expression, partially mediated by the transfer of miRNA-16 [24]. However, in contrast, another study demonstrated that exosomes released by BMSCs enhanced *VEGF* expression in gastric tumor cells by activating the extracellular signal-regulated kinase1/2 (ERK1/2) pathway [25].

Interestingly, we observed similar pro-angiogenic effects with BMSCs-derived exosomes, which induce higher ROS production in PC3 cells and, in consequence, an increase in the angiogenic potential. In this sense, our results strongly suggest that differences in the exosomal cargo of MSCs-derived exosomes might account for the observed opposing effects. It has been shown that MSCs from bone marrow (BMSCs) and adipose tissue (ATMSCs) secrete exosomes enriched with distinct miRNA and tRNA species, which deliver different information into their microenvironments [26]. In fact, Del Fattore and colleagues demonstrated differential effects of extracellular vesicles secreted by BMSCs and ATMSCs on glioblastoma cell proliferation, although no molecule responsible for these opposite effects was identified [50]. Indeed, all these contradictory observations demonstrate the complexity of the proteomic and genomic compositions of the exosomes and, hence, highlight the need to perform more studies to define the exact molecular constituents responsible for their biological effect. Also, it will be interesting to understand the physiological role of exosomes secreted in the endometrium environment and their role in controlling any malignant formation in their natural niche.

The anti-angiogenic effect of MenSCs-secreted exosomes was also studied on different types of tumor. While pancreatic cancer cell lines were not affected by the exosome treatment, both breast cancer cell lines presented a reduction in their angiogenic properties. The treatment with exosomes provoked a reduction in the VEGF level mediated by the lower ROS production induced by these extracellular vesicles. In consequence, we observed an inhibition in the angiogenic properties of the breast tumor cell-derived secretome, which opens the door for future investigation in exosome-based treatment against breast cancer. However, the fact that MenSCs-secreted exosomes differentially affect the tumor cell-derived angiogenic-secretome indicate, once again, the importance of performing further studies to better understand how exosomes affect different intracellular cascades and the expression of diverse genes.

Interestingly, we demonstrate here that the anti-angiogenic effect induced by MenSCs-secreted exosomes on prostate cancer cells is a unique property of the exosomes that cannot be extrapolated to their cells of origin, nor the MenSCs-derived secretome, or its microvesicular fraction. These results suggest that MenSCs secrete an antagonistic panel of paracrine factors where the concluding effect will depend on the stoichiometric ratio of pro- and anti-angiogenic factors. In contrast, its exosomes enclose a more homogenous population of molecules that together inhibit the prostate tumor cell-derived angiogenic-secretome. In line with this observation, our group have recently reported the superior effect of MenSCs-derived exosomes on the growth of cerebrocortical neurites in comparison with the MenSCs-derived microvesicles [34].

One limitation in the presented results concerns the doses of exosomes used in our experimental design. A kinetics experiment to define a maximal angiogenic response would be vital to define a possible translational strategy. Likewise, identifying the integral molecular constituents of the MenSCs-derived exosomes behind the observed effects will be very relevant in tuning or enhancing their potential. This would help to understand the specific intracellular pathways that are being affected by exosomes with the purpose of revealing their mechanism of action. Additionally, analysis of proteins and peptides by mass spectrometry and deep sequencing analysis of the miRNA profile of the exosomes secreted by different MSCs sources or batches of MenSCs will help us to reveal an inter- and intra-variability of the exosomes that would explain the opposing angiogenic effects described here and previously [24, 25]. Finally, it is important to note that in comparison with cells the exosomes present advantages, including higher stability, present no risk of aneuploidy, a lower possibility of immune rejection following *in vivo* allogeneic administration, are resistant to possible negative influence of the tumor environment and therefore, may provide an alternative or complementary therapy for various diseases [51].

## MATERIALS AND METHODS

### Biological samples and cell culture

This study was revised and approved by the Ethics Committee of the Universidad de los Andes. Menstrual fluids and bone marrow were obtained after informed consents following institutional guidelines from four healthy donors aged 24-38 years-old and two hip-operated patients aged 60-68 years-old, respectively [29, 31]. MSCs were isolated, cultured, and characterized as described previously [29, 31, 32], and cryopreserved at low passage (< 5) until use. Human umbilical vein endothelial cells (HUVEC), human pancreas adenocarcinoma MIA PaCa-2, human pancreas carcinoma PANC-1, metastatic human breast cancer MD-MB-231, metastatic human breast cancer MCF-7, metastatic human prostate adenocarcinoma PC3, metastatic human prostate carcinoma DU 145, metastatic human prostate carcinoma LNCaP and metastatic human prostate cancer VCaP cell lines were obtained from the American Type Culture Collection (ATCC, Manassas, VA, USA). HUVEC were cultured in endothelial growth media (EGM-2, Lonza, USA) with 5% FBS (Lonza, Walkersville, MD USA), 1% P/S (Life Technologies, Santiago, RM, Chile) and 1% L-glutamine (Life Technologies, Santiago, RM, Chile). MIA PaCa-2, PANC-1, MDA MB 231, PC3 and VCaP cell lines were cultured in DMEM (Gibco, Paisley, USA) with 10% FBS, 1% P/S and 1% L-glutamine. LNCaP, DU 145 and MCF7 cell lines were cultured in RPMI-1640 (Hyclone, GE Healthcare, Utah, USA) with 10% FBS, 1% P/S and 1% L-glutamine. All cells were maintained in a humidified incubator (37°C; 5% CO_2_) and routinely tested for the presence of mycoplasma (EZ-PCR Mycoplasma test kit, Biological Industries, Israel Beit-Haemek Ltd).

### Exosomes and microvesicles purification, characterization and internalization assay

MSCs primary cultures (3×10^7^ cells) were supplemented with serum-free DMEM containing 1% L-glutamine and 1% P/S (hereafter referred as serum-free DMEM), and incubated for 72 hours (h) at 37°C. Exosomes and microvesicles (MVs) were purified as previously described [33, 34]. Protein concentration was quantified by Bradford assay (BioRad, CA, USA). Lysed exosomes were used in all experiments as negative control. Exosomes were lysed with 1% triton X100, incubated with 0.5% trypsin for 30 min at 37°C and ultracentrifuged at 100,000 g for 70 min. Detailed protocols of exosomes characterization can be found in the supplemental online data.

To study the uptake of isolated exosomes, exosomes (20 μg) resuspended in phosphate buffered saline (PBS) were incubated with anti-CD63-FITC (Santa Cruz Biotechnology, CA, USA) or IgG1-FITC (isotype control; Biolegend, CA, USA) overnight at 4°C. The mix was pelleted by ultracentrifugation on a 30% sucrose/D_2_O density cushion (100,000 g for 70 min at 4°C) and the immuno-labeled exosomes were purified from the non-bound immunocomplexes. The exosomes interface was recovered and subjected to ultracentrifugation after dilution with PBS. Finally, the pellet containing immunofluorescent-labeled exosomes was resuspended in 200 μL PBS and added to 2×10^4^ PC3 cells for 3 hours at either 37°C or 4°C. Internalization of exosomes in PC3 cells was determined by fluorescence-activated cell sorting (FACS) (FACS Canto II, BD Biosciences, USA) and visualized with confocal microscopy (FV1200 Laser Scanning Microscope in combination with an IX83 automated inverted microscope platform, Olympus). For FACS analysis, cells were washed twice with PBS before acquisition in the flow cytometer; the data obtained was analyzed with FlowJo Software vX 10.0.7 (Tree star Inc, Stanford). For confocal microscopy, cells were washed twice with PBS and incubated with 2 μM CellTracker™ Red CMTPX (Life Technologies, Carlsbad, CA), according to the manufacturer's protocol. Cells were washed in PBS, fixed in 4% paraformaldehyde and mounted in Vectashield (Vector Laboratories Inc., Burlingame, CA) with 4,′6′-diaminido-2-phenylindole (DAPI; Bio Rad, USA). Images were taken with a 100 x objective of numerical aperture 1.4.

### Co-culture of cancer cells with MenSCs or their exosomes, microvesicles or secretome

For exosomes or MVs experiments, cancer cells (2×10^5^) were plated in a 6-well plate in complete media for 16 h. Semi-confluent layers of cancer cells were twice washed with PBS and serum-free DMEM was added prior to incubation with 10 μg of exosomes or MVs, lysed exosomes and vehicle, at 37°C for 36 h. Subsequently, the conditioned medium (CM) and cells were collected, and processed for further studies as required. For ROS and angiogenesis *in vitro* studies, cancer cells were incubated with 10 mM N-acetylcysteine (NAC) (Calbiochem), 250 μM H_2_O_2_ (Merck, Darmstadt, Germany) or exosomes plus H_2_O_2_.

For MenSCs co-cultured with cancer cells, PC3 cells (4×10^6^) were grown with MenSCs (2×10^6^) in complete media for 16 h. After two washes with PBS, cells were co-cultured with serum-free DMEM for 36 h, then separated by fluorescence-activated cell sorting (FACSAria, BD, USA) using double-staining for CD73-PECy7 and CD90-APC (BD Pharmingen, CA, USA). Sorted PC3 cells CD73^−^/CD90^−^ were processed for further studies as required. To study the effect of the MenSCs secretome on cancer cells, PC3 cells (4×10^6^) and MenSCs (2×10^6^) were co-cultured for 36 h separated by a 0.4 μm microporous membrane (Transwell, BD Bioscience) in serum-free DMEM. Cells were collected and processed for further studies as required.

### RNA expression analysis by RT-qPCR

Total RNAs were extracted using the RNeasy mini kit (Qiagen, CA, USA) and complementary cDNA was synthesized using 2 μg of RNA in a 20 μl reaction mixture using SuperScript III First-Strand Synthesis for RT-PCR (Invitrogen, Carlsbad, CA). RT-qPCR was performed using SYBR GREEN Reagents (Brilliant® II SYBR® Green QPCR Master Mix, Agilent Technologies). All primers sets were previously screened for efficiency and their sequences were *VEGF-A* (F): 5′ACACATTGTTGGAAGCAGCCC-3′, (R): 5′-AGGAAGGTCAACCACTCACACACA-3′; *bFGF* (F): 5′-AGAAGAGCGACCCTCACATCA-3′, (R): 5′-CGGT TAGCACACACTCCTTTG-3′; *GAPDH* (F): 5′-GGTC TCCTCTGACTTGAACA-3′, (R): 5′-GTGAGGGTCTCT CTCTTCCT-3′. All values were normalized to *GAPDH* housekeeping gene and expressed as relative expression or fold change using the respective formulae 2^− ΔΔCt^.

### Western blot analysis of VEGF

Equal amounts of proteins were resolved by sodium dodecyl sulfate–polyacrylamide gel electrophoresis and blotted, and membranes were first probed with anti-VEGF antibody (rabbit polyclonal 1:2000, Abcam) and reprobed with β-actin antibody (mouse monoclonal 1:1000, Santa Cruz). Goat anti-mouse HRP and goat anti-rabbit HRP (BioRad) were used as secondary antibodies. Western blots were revealed by enhanced chemiluminescence (Amersham). Scanned bands were quantified using ImageJ software Version 1.43 (National Institutes of Health, http://rsb.info.nih.gov/ij/). All Western blot results were evaluated by densitometric scanning, corrected with respect to β-actin expression, and expressed relative to the value obtained with the corresponding control (arbitrarily set as 1). Equal protein loading was assessed by anti–β-actin immunoblotting.

### ELISA assay

Conditioned media from PC3 cells (PC3-CM) treated with exosomes, lysed exosomes or untreated, were collected after 36 hours incubation. For detection of VEGF, CM was concentrated approximately 50 fold using ultracentrifugation units (Amicon Ultra; Millipore, Tullagreen, IRL) with a 3KDa molecular mass cut-off according to the manufacturer′s instructions. VEGF was then detected by ELISA (Human VEGF Duoset; R&D system, Minneapolis, MN) according to the manufacturer's recommended protocol.

### Luciferase reporter assay for NF-kappaB (NF-κB) activity

PC3 cells were transfected with plasmid DNA using the Lipofectamine 2000 transfection reagent (Invitrogen, CA, USA). Briefly, 3×10^4^ cells were plated onto each well of 24-well plates. After 24 hours, cells were transfected with NF-kB-luciferase reporter plasmid (1 μg) using Opti-MEM I Reduced Serum Medium (Invitrogen, CA, USA), and 6 hours later the medium was replaced with complete media. Twenty-four hours post transfection, cells were cultured with or without MenSCs-derived exosomes. Cells were cultured for another 24 hours and harvested for a luciferase assay (Promega, Madison, WI). In all experiments, activities of firefly luciferase were measured using the Promega Luciferase reporter system, and expressed as relative luciferase light units (RLU). The data was normalized by protein concentration.

### ROS measurement

Reactive oxygen species (ROS) were detected by the DCF method. Briefly, 8×10^4^ cancer cells were cultured in a 24-well plate overnight. Cells were loaded with 10 μM 2′, 7′-dichlorofluorescin diacetate (DCFDA; Sigma-Aldrich, St. Louis MO, USA) with or without 10 μg exosomes for 2 h at 37°C. A positive and negative control were also set up using 10 mM NAC and 250 μM H_2_O_2_, respectively. Cells were acquired using a FACS Canto II Flow cytometer and analyzed on FlowJo Software vX 10.0.7.

### *In vitro* tube formation assay

Matrigel grow factor reduced matrix (250 μl; 354235 BD Bioscience, USA) was added to 24-well plates and allowed to polymerize at 37°C for 30 minutes. HUVEC cells (6×10^4^) were resuspended in 250 μl of CM from cancer cells treated with exosomes (−CM^EXO^), exosomes plus H_2_O_2_ (−CM^EXO^+H_2_O_2_), lysed exosomes (−CM^LysEXO^), NAC (−CM+NAC), or untreated (−CM) before seeded on polymerized matrigel. As negative and positive controls, 250 μl of DMEM and EGM-2 (Lonza, Walkersville, MD) were used, respectively. After 5 h, tube formation was examined with a phase-contrast microscope and 5 representative images per well were captured using an Olympus U-RFL-T camera. Quantification of tube formation was analyzed using WimTube software (Wimasis GmbH, Munich, Germany).

### Animal studies

All mouse studies were performed at the animal facility of the Universidad de los Andes, in accordance with protocols revised and approved by the Institutional Animal Care and Use Committee of Universidad de los Andes. NOD.Cg-Prkdcscid Il2rgtm1Wjl/SzJ (NSG) mice were purchased from Jackson Laboratories (Bar Harbor, ME, USA).

For matrigel plug assay, 8-week-old mice were randomly divided into 5 groups (n=4-12 plugs per group). All groups were implanted subcutaneously with a mixture of 250 μl of growth factor-reduced matrigel (BD Bioscience, San Jose, CA, USA) and 4×10^6^ HUVEC cells previously resuspended in 250 μl of DMEM alone or EGM-2, or the PC3-CM previously treated with exosomes (PC3-CM^EXO^), lysed exosomes (PC3-CM^LysEXO^), or untreated (PC3-CM). After 14 days, matrigel plugs were harvested and processed for hemoglobin quantification as previously described [35]. The number of blood vessels was counted using ImageJ software (NIH, Bethesda, MD, USA).

For mouse prostate tumor growth, subcutaneous PC3 tumor xenografts were established by injection of 1.5×10^6^ cells into the flanks of 10-week-old male mice (n=8-14 tumors per group). Once the mean tumor volume reached 80 mm^3^, mice were intratumorally injected with vehicle (100μL PBS/tumor) or exosomes (10μg/100μL/tumor) or lysed exosomes (10μg/100μL/tumor) 3 times with 3-days intervals. Tumor size was recorded every 2- to 3-day intervals and the tumor growth was calculated as previously described [36]. After 12 days, tumor samples were recovered, photographed, and weighed. For histopathologic analysis, tumor tissues were fixed with 10% formalin, and CD31, VEGF and FGF immunostaining was performed (CyS Laboratory, Santiago, Chile) (detailed protocol in supplementary methods). Hemoglobin concentration was performed in 20 mg of tumor tissue as previously described [35].

### Statistical analysis

One-way ANOVA followed by Tukey's post-test was used for analysis of multiple comparison groups. Two-tailed Student's unpaired t-test was used to compare two groups. Statistical analyses were performed using GraphPad Prism 5 (GraphPad Software, Inc., San Diego, CA, USA). The numbers of samples per group (n) are specified in the figure legends. Statistical significance was set at *p < 0.05; **p < 0.01; ***p < 0.001.

## CONCLUSION

Here, we demonstrate that MenSCs-derived exosomes possess an anti-tumoral activity in prostate cancer by blocking tumor-induced angiogenesis in a ROS-dependent mechanism. MenSCs alone or their secretome including MVs failed to achieve a similar effect. This activity was also specific to the menstrual cells source as opposite effects were observed when BMSCs were used. Moreover, the anti-angiogenic effect was also dependent on the type of cancer cell lines used. These results highlight the need to carefully single out the appropriate source of MSCs and the type of cancer to be treated in order to obtain the desired outcome. These results pave the way for novel anti-angiogenic tumor treatments based on exosomes secreted by MenSCs, although further studies are needed before the true contribution of MenSCs-derived exosomes to the clinical scenario.

## SUPPLEMENTARY DATA FIGURES



## Abbreviations

BMSCs: bone marrow derived mesenchymal stem cells
CM: conditioned media
Exo: exosomes
bFGF: basic fibroblast growth factor
MSCs: mesenchymal stem cells
MenSCs: menstrual derived mesenchymal stem cells
NFκB: nuclear factor kappa B
ROS: reactive oxygen species
VEGF: vascular endothelial growth factor

## References

[1] Koch S, Tugues S, Li X, Gualandi L, Claesson-Welsh L (2011). Signal transduction by vascular endothelial growth factor receptors. The Biochemical journal.

[2] Kim YW, Byzova TV (2014). Oxidative stress in angiogenesis and vascular disease. Blood.

[3] Wang Y, Zang QS, Liu Z, Wu Q, Maass D, Dulan G, Shaul PW, Melito L, Frantz DE, Kilgore JA, Williams NS, Terada LS, Nwariaku FE (2011). Regulation of VEGF-induced endothelial cell migration by mitochondrial reactive oxygen species. American journal of physiology Cell physiology.

[4] Szatrowski TP, Nathan CF (1991). Production of large amounts of hydrogen peroxide by human tumor cells. Cancer research.

[5] Xia C, Meng Q, Liu LZ, Rojanasakul Y, Wang XR, Jiang BH (2007). Reactive oxygen species regulate angiogenesis and tumor growth through vascular endothelial growth factor. Cancer research.

[6] Kim J, Koyanagi T, Mochly-Rosen D (2011). PKCdelta activation mediates angiogenesis via NADPH oxidase activity in PC-3 prostate cancer cells. The Prostate.

[7] Studeny M, Marini FC, Dembinski JL, Zompetta C, Cabreira-Hansen M, Bekele BN, Champlin RE, Andreeff M (2004). Mesenchymal stem cells: potential precursors for tumor stroma and targeted-delivery vehicles for anticancer agents. Journal of the National Cancer Institute.

[8] Kucerova L, Altanerova V, Matuskova M, Tyciakova S, Altaner C (2007). Adipose tissue-derived human mesenchymal stem cells mediated prodrug cancer gene therapy. Cancer research.

[9] Zhang T, Lee YW, Rui YF, Cheng TY, Jiang XH, Li G (2013). Bone marrow-derived mesenchymal stem cells promote growth, angiogenesis of breast and prostate tumors. Stem cell research & therapy.

[10] Beckermann BM, Kallifatidis G, Groth A, Frommhold D, Apel A, Mattern J, Salnikov AV, Moldenhauer G, Wagner W, Diehlmann A, Saffrich R, Schubert M, Ho AD, Giese N, Buchler MW, Friess H (2008). VEGF expression by mesenchymal stem cells contributes to angiogenesis in pancreatic carcinoma. British journal of cancer.

[11] Orecchioni S, Gregato G, Martin-Padura I, Reggiani F, Braidotti P, Mancuso P, Calleri A, Quarna J, Marighetti P, Aldeni C, Pruneri G, Martella S, Manconi A, Petit JY, Rietjens M, Bertolini F (2013). Complementary populations of human adipose CD34+ progenitor cells promote growth, angiogenesis, and metastasis of breast cancer. Cancer research.

[12] Keramidas M, de Fraipont F, Karageorgis A, Moisan A, Persoons V, Richard MJ, Coll JL, Rome C (2013). The dual effect of mesenchymal stem cells on tumour growth, tumour angiogenesis. Stem cell research & therapy.

[13] Otsu K, Das S, Houser SD, Quadri SK, Bhattacharya S, Bhattacharya J (2009). Concentration-dependent inhibition of angiogenesis by mesenchymal stem cells. Blood.

[14] Ho IA, Toh HC, Ng WH, Teo YL, Guo CM, Hui KM, Lam PY (2013). Human bone marrow-derived mesenchymal stem cells suppress human glioma growth through inhibition of angiogenesis. Stem cells.

[15] Alcayaga-Miranda F, Varas-Godoy M, Khoury M (2016). Harnessing the Angiogenic Potential of Stem Cell-Derived Exosomes for Vascular Regeneration. Stem cells international.

[16] Akimoto K, Kimura K, Nagano M, Takano S, To'a Salazar G, Yamashita T, Ohneda O (2013). Umbilical cord blood-derived mesenchymal stem cells inhibit, but adipose tissue-derived mesenchymal stem cells promote, glioblastoma multiforme proliferation. Stem cells and development.

[17] Cousin B, Ravet E, Poglio S, De Toni F, Bertuzzi M, Lulka H, Touil I, Andre M, Grolleau JL, Peron JM, Chavoin JP, Bourin P, Penicaud L, Casteilla L, Buscail L, Cordelier P (2009). Adult stromal cells derived from human adipose tissue provoke pancreatic cancer cell death both in vitro and in vivo. PloS one.

[18] Klopp AH, Gupta A, Spaeth E, Andreeff M, Marini F (2011). Concise review: Dissecting a discrepancy in the literature: do mesenchymal stem cells support or suppress tumor growth?. Stem cells.

[19] Menge T, Gerber M, Wataha K, Reid W, Guha S, Cox CS, Dash P, Reitz MS, Khakoo AY, Pati S (2013). Human mesenchymal stem cells inhibit endothelial proliferation and angiogenesis via cell-cell contact through modulation of the VE-Cadherin/beta-catenin signaling pathway. Stem cells and development.

[20] S ELA, Mager I, Breakefield XO, Wood MJ (2013). Extracellular vesicles: biology and emerging therapeutic opportunities. Nature reviews Drug discovery.

[21] Costa-Silva B, Aiello NM, Ocean AJ, Singh S, Zhang H, Thakur BK, Becker A, Hoshino A, Mark MT, Molina H, Xiang J, Zhang T, Theilen TM, Garcia-Santos G, Williams C, Ararso Y (2015). Pancreatic cancer exosomes initiate pre-metastatic niche formation in the liver. Nature cell biology.

[22] Mulcahy LA, Pink RC, Carter DR (2014). Routes, mechanisms of extracellular vesicle uptake. Journal of extracellular vesicles.

[23] Urbich C, Kuehbacher A, Dimmeler S (2008). Role of microRNAs in vascular diseases, inflammation, and angiogenesis. Cardiovascular research.

[24] Lee JK, Park SR, Jung BK, Jeon YK, Lee YS, Kim MK, Kim YG, Jang JY, Kim CW (2013). Exosomes derived from mesenchymal stem cells suppress angiogenesis by down-regulating VEGF expression in breast cancer cells. PloS one.

[25] Zhu W, Huang L, Li Y, Zhang X, Gu J, Yan Y, Xu X, Wang M, Qian H, Xu W (2012). Exosomes derived from human bone marrow mesenchymal stem cells promote tumor growth in vivo. Cancer letters.

[26] Baglio SR, Rooijers K, Koppers-Lalic D, Verweij FJ, Perez Lanzon M, Zini N, Naaijkens B, Perut F, Niessen HW, Baldini N, Pegtel DM (2015). Human bone marrow- and adipose-mesenchymal stem cells secrete exosomes enriched in distinctive miRNA and tRNA species. Stem cell research & therapy.

[27] Pepper MS (1997). Manipulating angiogenesis. From basic science to the bedside. Arteriosclerosis, thrombosis, and vascular biology.

[28] Maas JW, Groothuis PG, Dunselman GA, de Goeij AF, Struyker Boudier HA, Evers JL (2001). Endometrial angiogenesis throughout the human menstrual cycle. Human reproduction.

[29] Alcayaga-Miranda F, Cuenca J, Luz-Crawford P, Aguila-Diaz C, Fernandez A, Figueroa FE, Khoury M (2015). Characterization of menstrual stem cells: angiogenic effect, migration, hematopoietic stem cell support in comparison with bone marrow mesenchymal stem cells. Stem cell research & therapy.

[30] Khoury M, Alcayaga-Miranda F, Illanes SE, Figueroa FE (2014). The promising potential of menstrual stem cells for antenatal diagnosis, cell therapy. Frontiers in immunology.

[31] Luz-Crawford P, Torres MJ, Noel D, Fernandez A, Toupet K, Alcayaga-Miranda F, Tejedor G, Jorgensen C, Illanes SE, Figueroa FE, Djouad F, Khoury M (2016). The immunosuppressive signature of menstrual blood mesenchymal stem cells entails opposite effects on experimental arthritis and graft versus host diseases. Stem cells.

[32] Alcayaga-Miranda F, Cuenca J, Martin A, Contreras L, Figueroa FE, Khoury M (2015). Combination therapy of menstrual derived mesenchymal stem cells and antibiotics ameliorates survival in sepsis. Stem cell research & therapy.

[33] Thery C, Amigorena S, Raposo G, Clayton A (2006). Isolation and characterization of exosomes from cell culture supernatants and biological fluids. Current protocols in cell biology / editorial board, Juan S Bonifacino.

[34] Lopez-Verrilli MA, Caviedes A, Cabrera A, Sandoval S, Wyneken U, Khoury M (2016). Mesenchymal stem cell-derived exosomes from different sources selectively promote neuritic outgrowth. Neuroscience.

[35] Gonzalez PL, Carvajal C, Cuenca J, Alcayaga-Miranda F, Figueroa FE, Bartolucci J, Salazar-Aravena L, Khoury M (2015). Chorion Mesenchymal Stem Cells Show Superior Differentiation, Immunosuppressive, and Angiogenic Potentials in Comparison With Haploidentical Maternal Placental Cells. Stem cells translational medicine.

[36] Alcayaga-Miranda F, Cascallo M, Rojas JJ, Pastor J, Alemany R (2010). Osteosarcoma cells as carriers to allow antitumor activity of canine oncolytic adenovirus in the presence of neutralizing antibodies. Cancer gene therapy.

[37] Meng X, Ichim TE, Zhong J, Rogers A, Yin Z, Jackson J, Wang H, Ge W, Bogin V, Chan KW, Thebaud B, Riordan NH (2007). Endometrial regenerative cells: a novel stem cell population. Journal of translational medicine.

[38] Lopez-Verrilli MA, Picou F, Court FA (2013). Schwann cell-derived exosomes enhance axonal regeneration in the peripheral nervous system. Glia.

[39] Escrevente C, Keller S, Altevogt P, Costa J (2011). Interaction and uptake of exosomes by ovarian cancer cells. BMC cancer.

[40] Svensson KJ, Christianson HC, Wittrup A, Bourseau-Guilmain E, Lindqvist E, Svensson LM, Morgelin M, Belting M (2013). Exosome uptake depends on ERK1/2-heat shock protein 27 signaling and lipid Raft-mediated endocytosis negatively regulated by caveolin-1. The Journal of biological chemistry.

[41] Huang S, Pettaway CA, Uehara H, Bucana CD, Fidler IJ (2001). Blockade of NF-kappaB activity in human prostate cancer cells is associated with suppression of angiogenesis, invasion, and metastasis. Oncogene.

[42] Kumar B, Koul S, Khandrika L, Meacham RB, Koul HK (2008). Oxidative stress is inherent in prostate cancer cells and is required for aggressive phenotype. Cancer research.

[43] Deer EL, Gonzalez-Hernandez J, Coursen JD, Shea JE, Ngatia J, Scaife CL, Firpo MA, Mulvihill SJ (2010). Phenotype and genotype of pancreatic cancer cell lines. Pancreas.

[44] Xu W, Liu LZ, Loizidou M, Ahmed M, Charles IG (2002). The role of nitric oxide in cancer. Cell research.

[45] Fujioka S, Sclabas GM, Schmidt C, Frederick WA, Dong QG, Abbruzzese JL, Evans DB, Baker C, Chiao PJ (2003). Function of nuclear factor kappaB in pancreatic cancer metastasis. Clin Cancer Res.

[46] Qiao L, Zhao TJ, Wang FZ, Shan CL, Ye LH, Zhang XD (2008). NF-kappaB downregulation may be involved the depression of tumor cell proliferation mediated by human mesenchymal stem cells. Acta pharmacologica Sinica.

[47] Laurent A, Nicco C, Chereau C, Goulvestre C, Alexandre J, Alves A, Levy E, Goldwasser F, Panis Y, Soubrane O, Weill B, Batteux F (2005). Controlling tumor growth by modulating endogenous production of reactive oxygen species. Cancer research.

[48] Wang Y, Huang X, Cang H, Gao F, Yamamoto T, Osaki T, Yi J (2007). The endogenous reactive oxygen species promote NF-kappaB activation by targeting on activation of NF-kappaB-inducing kinase in oral squamous carcinoma cells. Free radical research.

[49] Kim YW, West XZ, Byzova TV (2013). Inflammation and oxidative stress in angiogenesis and vascular disease. Journal of molecular medicine.

[50] Del Fattore A, Luciano R, Saracino R, Battafarano G, Rizzo C, Pascucci L, Alessandri G, Pessina A, Perrotta A, Fierabracci A, Muraca M (2015). Differential effects of extracellular vesicles secreted by mesenchymal stem cells from different sources on glioblastoma cells. Expert opinion on biological therapy.

[51] Yu B, Zhang X, Li X (2014). Exosomes derived from mesenchymal stem cells. International journal of molecular sciences.

