# Supplementary material for: Prostate tumor-induced angiogenesis is blocked by exosomes derived from menstrual stem cells through the inhibition of reactive oxygen species
Source: Oncotarget. 2016 Jun 6;7(28):44462–77. doi: 10.18632/oncotarget.9852 (PMC5190111; doi:10.18632/oncotarget.9852)
Supplement: Supplementary file 1 [file oncotarget-07-44462-s001.pdf]

Prostate tumor-induced angiogenesis is blocked by exosomes derived from menstrual stem cells through the inhibition of reactive oxygen species

SUPPLEMENTARY DATA

Tri-lineage differentiation

All cells were evaluated in their capacity to differentiation to adipocytes, osteocytes and chondrocytes using the StemPro® Differentiation Kits (Gibco, Carlsbad, CA, USA) according to the manufacturer's instruction. After 14-21 days, adipogenic, osteogenic and chondrogenic differentiation of stem cells were determined using Oil Red O (Sigma–Aldrich, USA), Alizarin Red (Sigma–Aldrich, USA) and Safranin O (Merck) staining, respectively.

Immunophenotypic characterization of MenSCs

Immunophenotyping was performed by fluorescence-activated cell sorting (FACS) using a FACSCanto II cytometer (BD Biosciences, San Jose, CA, USA) after staining with monoclonal antibodies CD105, CD90, CD73, CD44, HLA-ABC, HLA-DR, CD34 and CD45 (all from BD Pharmingen San Jose, CA, USA) using standard protocol. In brief, cells were harvested, washed with a cytometry buffer (PBS + 0.2% BSA + 0.01% Sodium Azide [all from Sigma-Aldrich, St Lois, USA]) and incubated with the specific labelled antibodies in cytometer buffer for 20 min at 4°C. In all experiments, matching isotype antibodies were used as negative controls. In addition, LIVE/DEAD®Fixable dead cell stain kit (Invitrogen) was used to determine the viability cells by flow cytometry according to the manufacturer's protocol. Data (5,000 events) were analyzed on FlowJo Software vX 10.0.7 (Tree star Inc, Stanford).

Exosomes characterization

Exosome size and shape were evaluated by electron microscopy (EM). Briefly, 30 µg exosomes were fixed with 2% PFA and deposited on Formvar-carbon-coated EM grids, and contrasted with uranyl acetate. All the grids were examined with a Philips Tecnai 12 electron microscope operated at 80 kV. Nanoparticle tracking analysis (NTA) were performed using a NanoSight NS500 instrument (NanoSight NTA 2.3 Nanoparticle Tracking and Analysis Release Version Build 0033) following the manufacturer's instructions. Briefly, exosomes fractions were processed in duplicate and diluted with PBS over a range of concentration to obtain between 10 and 100 particles per image. Exosomes samples were mixed before analysis into the chamber and two videos per sample were processed and analyzed to give the mean, mode, and median particle size together with an estimated number of particles.

Exosome markers were identified by Western blot according to previously published methods (1). In brief, exosomal proteins (15µg) were separated on a 12% polyacrylamide gel and transferred to polyvinylidene difluoride membrane (PVDF; Thermo Scientific) for 1 hour at 100 V. The membrane was washed in wash buffer (PBS TWEEN 0.1%) three times for 10 min and blocked with 5% skimmed milk in PBS TWEEN (0.1%) for one hour at room temperature under agitation. The blocked membrane was further probed with the previously identified exosome-specific marker anti-CD63 (rabbit polyclonal 1:500; Santa Cruz Biotechnology), anti-heat shock protein 70 (Hsp70, mouse monoclonal 1:500, Stressgen), anti-heat shock protein 90 (Hsp90, rabbit polyclonal 1:500, Stressgen), and anti-cytochrome C (mouse monoclonal 1:1000, BD Bioscience). This was achieved by incubating the membrane in primary antibody diluted in 5% skim milk in PBS TWEEN (0.1%) at 4°C overnight on the laboratory rocker. After an overnight incubation, the membrane was washed with wash buffer and exposed to the appropriated secondary antibody (goat anti-rabbit HRP or goat anti-mouse HRP; BioRad). The membrane was washed 3 times for 10 min in wash buffer. Blots were revealed by the enhanced chemiluminescence method (Amersham). Protein loading was evaluated by SDS-PAGE followed by silver staining.

Immunohistochemistry

For immunohistochemistry analysis, tumor samples fixed in formalin were embedded in paraffin following standard histopathologic methods using Tissue-Tek®TEC Embedding Console System (Sakura, Tokyo, Japan). Tissue sections of 5-µm thickness were cut on an Accu-Cut®SRM microtome (Sakura, Tokyo, Japan), incubated overnight at 65°C, de-paraffinized and rehydrated with xylene and ethanol according to standard procedures. Immunodetection was performed after inhibition endogenous peroxidase activity for 15 minutes in 0.3% H_2O_2 in methanol, heat antigen retrieval and blocked for 2 hours with 10% normal horse serum (Gibco) diluted in PBS. Tissue sections were incubated overnight at 4°C with anti-CD31 (Rabbit polyclonal 1:50, Abcam), anti-HIF-1-α (Mouse monoclonal 1:500, Abcam) and anti-VEGF

(Mouse monoclonal, 1:200, Dako, Germany). Slides were washed in PBS 0.2% Triton X-100 and incubated with peroxidase-conjugated EnVision (Dako, Germany). Finally, sections were developed with diaminobenzidine (Dako) and counterstained with hematoxylin. For evaluation of vascular density, slides were scanned in the light microscope at × 40 magnification. Vascular density was determined by counting the number of $CD31^+$ microvessels per mm^2 using an Olympus CX41 microscope. A microvessel was defined as any distinct $CD31^+$ cell cluster with or without a vessel lumen. Counting was performed at least in 8 different representative fields of each of the tumors.

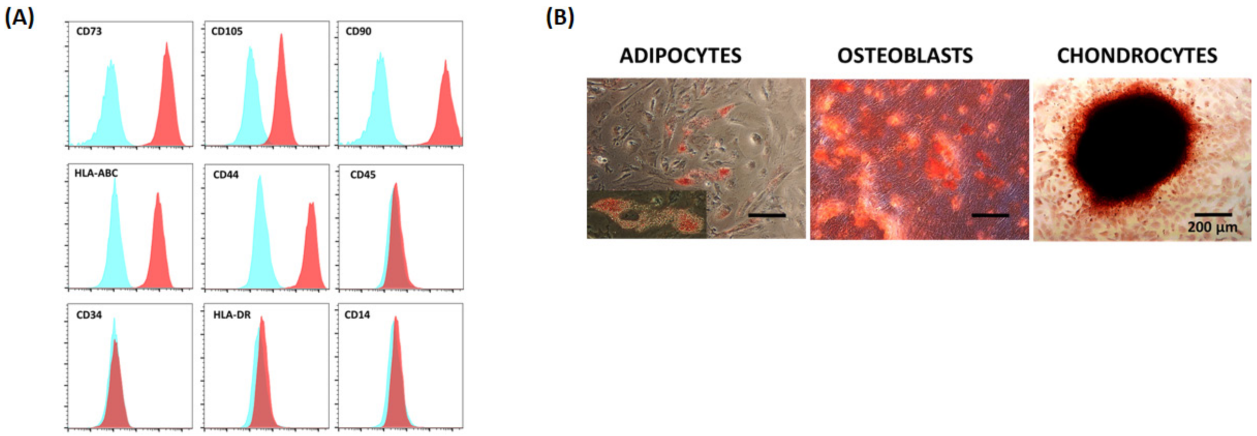

Supplementary Figure S1: Characterization of MenSCs. Stem cells isolated from the menstrual fluid were characterized according to ISCT guidlines. **A.** MenSCs showed stem cell-like immunophenotypic markers. FACS profile of a representative MenSCs sample. Red filled histograms denote the fluorescent profile of the indicated antigens and light-blue filled histograms correspond to isotype-matched controls. **B.** MenSCs displayed mesodermal differentiation. Tri-lineage differentiation of a representative MenSCs sample. Cells were cultured with adipogenic, osteogenic and chondrogenic induction media for 14-21 days and then stained with Oil Red O, Alizarin Red and Safranin O staining, respectively. Abbreviations: MenSCs, menstrual derived mesenchymal stem cells; ISCT, international society of cellular therapy. Scale Bar: 200µm.

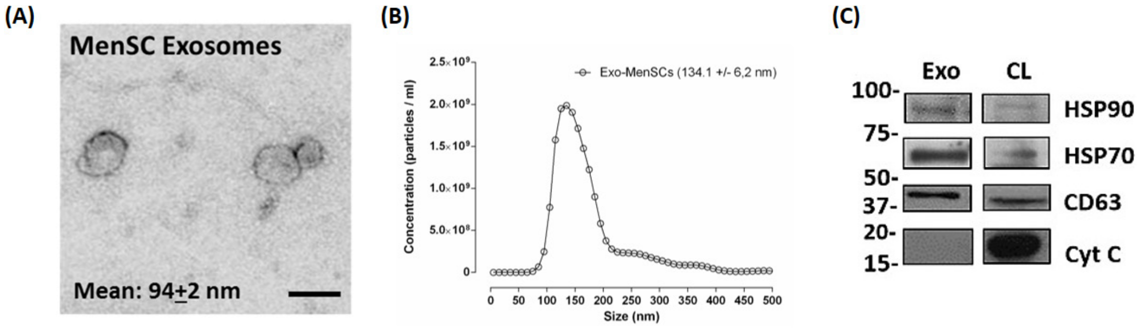

Supplementary Figure S2: Characterization of MenSCs-derived exosomes. Exosomes were purified from the conditioned media of MenSCs using differential centrifugation. **A.** Electron microscopy micrographs of exosomes isolated from MenSCs cell culture conditioned media. Scale Bar: 100 nm. **B.** Particle size by nanoparticle tracking analysis for MenSCs-secreted exosomes. Data are presented as mode values ± SE **C.** Western blot analysis of exosomes and cell lysates. 15 µg of proteins were loaded per lane. Abbreviations: MenSCs, menstrual derived mesenchymal stem cells; Exo, exosomes; CL, cell lysates; nm, nanometers; µg, micrograms; ml, milliliters; SE, standard error.

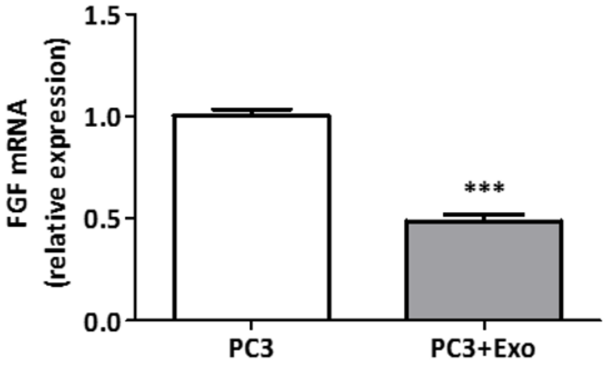

Supplementary Figure S3: *bFGF* expression in prostate PC3 cancer cell line after MenSCs-derived exosomes treatment. PC3 cancer cells were incubated in the absence or presence of MenSCs-derived exosomes for 36 hours and their effects on *bFGF* were determined. Relative expression levels of *bFGF* were assessed by qRT-PCR. Data are presented as mean values ± SE. Abbreviations: bFGF, basic fibroblast growth factor; MenSCs, menstrual derived mesenchymal stem cells; Exo, exosomes; ns, non-significant; SE, standard error.

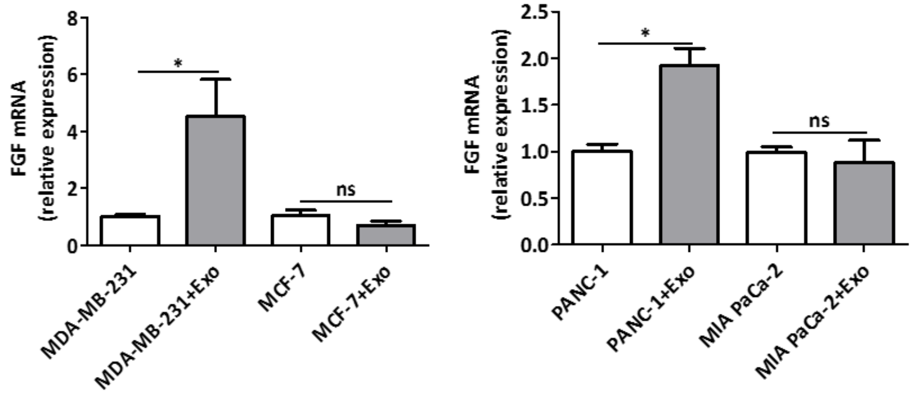

Supplementary Figure S4: *bFGF* expression in breast and pancreatic cancer cell lines after MenSCs-derived exosomes treatment. Breast (MDA-MB-231 and MCF-7) and pancreatic (MIA PaCa-2 and PANC-1) cancer cells were incubated in the absence or presence of MenSCs-derived exosomes for 36 hours and their effects on *bFGF* were determined. Relative expression levels of *bFGF* were assessed by qRT-PCR. Data are presented as mean values ± SE. Abbreviations: bFGF, basic fibroblast growth factor; MenSCs, menstrual derived mesenchymal stem cells; Exo, exosomes; ns, non-significant; SE, standard error.
